# Diagnostic Value of Spectral-Domain Optical Coherence Tomography for Polypoidal Choroidal Vasculopathy: A Systematic Review and Meta-Analysis

**DOI:** 10.3389/fmed.2022.878946

**Published:** 2022-06-15

**Authors:** Yang Jiang, Shixin Qi

**Affiliations:** ^1^Department of Ophthalmology, Tianjin Baodi Hospital, Tianjin, China; ^2^Baodi Clinical College, Tianjin Medical University, Tianjin, China

**Keywords:** spectral-domain optical coherence tomography, polypoidal choroidal vasculopathy, sensitivity, specificity, diagnostic value, meta-analysis

## Abstract

**Purpose:**

To evaluate the diagnostic value of spectral-domain optical coherence tomography (SD-OCT) for polypoidal choroidal vasculopathy (PCV).

**Methods:**

A search of electronic databases was conducted from 2010 to 2021 to review the relevant literature on SD-OCT to identify PCV and other lesions causing serious or serosanguinous retinal pigment epithelial detachment (PED), specifically neovascular age-related macular degeneration (nvAMD). The QUADAS-2 scale was used to evaluate the quality of the literature. We performed a meta-analysis, including heterogeneity tests, analyze and synthesize the study data, meta-regression analysis, subgroup analysis, Fagan's plot, sensitivity analysis and publication bias tests.

**Results:**

A total of 12 related studies involving 1,348 eyes were included in this study, and the random-effects model was used for meta-analysis. The results showed that the pooled sensitivity of SD-OCT in the diagnosis of PCV was 0.87 (95% CI: 0.84–0.89), the pooled specificity was 0.83 (95% CI: 0.80–0.86), and the pooled positive/negative likelihood ratios were 5.38 (95% CI: 3.28–8.80) and 0.16 (95% CI: 0.10–0.25), respectively. The diagnostic odds ratio (DOR) was 36.07 (95% CI: 15.98–81.40), and the area under the sROC curve (AUC) was 0.9429. When the pre–test probability was set at 20%, the post-test positive and negative probabilities were 58% and 4%, respectively. Meta-regression indicated that race was the primary source of heterogeneity (P <0.05). The Deeks' funnel plot showed no significant publication bias in this study (P>0.05).

**Conclusion:**

SD-OCT has high sensitivity and specificity for the diagnosis of PCV, as well as significant clinical applicability. Since color fundus photography (CFP) is more clinically available and can improve the diagnostic efficacy, we recommend SD-OCT combined with CFP to diagnose PCV, especially without indocyanine green angiography (ICGA).

**Systematic Review Registration:**

https://inplasy.com/inplasy-2021-12-0048/, identifier: INPLASY2021120048.

## Introduction

Polypoidal choroidal vasculopathy (PCV) is characterized by choroidal vascular abnormalities with or without abnormal branching vascular networks (BVNs), as shown by indocyanine green angiography (ICGA). The disease occurs most frequently in Asian populations (1, 2) and presents as a subretinal orange-red lesion in the fundus with recurrent serous or serosanguinous retinal pigment epithelial detachment (PED) (3). In the past, due to the lack of understanding of PCV and limitations of examination methods, many scholars considered it a subtype of neovascular age-related macular degeneration (nvAMD), which is characterized by type 1 aneurysmal choroidal neovascularization (4–6). However, there are significant differences between the two in terms of pathogenesis, clinical characteristics, natural course and prognosis, especially in terms of response to treatment. Photodynamic therapy (PDT) promotes regression or stabilization of choroidal polypoidal lesions and even improves visual acuity in patients with PCV (7), so it has a better therapeutic effect than in AMD (8). Anti-vascular endothelial growth factor (VEGF) drugs are the essential treatment for typical nvAMD. Although they effectively inhibit exudation caused by polypoidal lesions or abnormal BVNs, alleviate retinal edema, and improve visual acuity in PCV patients, they do not fundamentally degenerate polypoidal lesions and abnormal BVNs (2). Recently, the EVEREST II study (9), a 24-month phase IV double-blind, multicenter, randomized clinical test, recommended the combination of PDT and Anti-VEGF drugs to improve patients' vision. Therefore, it is very important to distinguish PCV and nvAMD in the initial diagnosis.

Currently, ICGA is the best visual diagnostic method for PCV, and it has been proven to be the gold standard for diagnosis (7). However, it is an invasive and expensive method, and ICGA is contraindicated in some patients due to impaired liver and kidney function, allergy to contrast agents, and pregnancy. In addition, a shortage of reagents or equipment in developing countries limits its clinical application (10). SD-OCT can clearly display the detailed structure of retinal cross-sections at the histological level. In addition, it is non-invasive, convenient, economical and non-contact, thus gaining increasing use in the diagnosis of PCV. Since De Salvo et al. (11) first diagnosed PCV with SD-OCT in 2014, other studies have used individual SD-OCT diagnostic strategies to diagnose PCV in different populations with varying sensitivity and specificity. At present, no studies have summarized and analyzed these data to draw a consistent conclusion. Therefore, we performed a systematic review and meta-analysis to explore the diagnostic value of SD-OCT for PCV.

## Materials and Methods

### Protocol and Registration

This systematic review and meta-analysis was designed and reported in accordance with the Preferred Reporting Items for Systematic Reviews and Meta-Analyses (PRISMA) (12). The protocol for this systematic review was registered with the International Platform of Registered Systematic Review and Meta-analysis Protocols (INPLASY) (registration number: INPLASY2021120048; https://inplasy.com/inplasy-2021-12-0048/).

### Search Strategy

We systematically searched electronic databases such as Pubmed, Embase, Cochrane, Web of Science, China National Knowledge Infrastructure (CNKI), Wanfang database (Wanfang Data), China Science and Technology Journal Database (VIP), and China Biomedical Literature on disc (CBMdisc) from 2010 to 2021. Relevant Chinese and English literature were searched. Database searches were performed using a combination of MeSH and keywords. The search keywords were “polypoidal choroidal vasculopathy,” “PCV,” “tomography, optical coherence,” “coherence tomography, optical,” “OCT tomography,” “tomography, OCT,” “optical coherence tomography,” “spectral-domain optical coherence tomography,” “spectral domain optical coherence tomography,” “SD-OCT,” “sensitivity and specificity,” “predictive value of tests,” and “accuracy.” Details of the search strategy are listed in the Supplementary Digital Content (Supplementary Appendix). One author (JY) performed the strategy, and the other author (QSX) reviewed the process independently. When the two authors disagreed, the decision was made through open consultation.

### Eligibility Criteria

The inclusion criteria for the published articles in this meta-analysis were as follows. (1) Study type: published trials related to PCV diagnosis by SD-OCT; (2) PCV cases confirmed by ICGA (polypoidal hyperfluorescence with or without abnormal BVNs on ICGA). Fundus fluorescein angiography (FFA) may be included as an additional diagnostic criterion; (3) SD-OCT examination or any angiography used to identify serous or serosanguinous PED in the macular area involving nvAMD, central serous chorioretinopathy (CSC), occult or idiopathic choroidal neovascularization (occult or idiopathic CNV), or retinal angiomatous proliferation (RAP).

Exclusion criteria were defined as the following. (1) Basic investigations such as animal experiments; (2) Reviews, systematic reviews, and meta-analyses; (3) Literature without extractable data items of the fourfold table: true positive (TP), false positive (FP), true negative (TN), and false negative (FN); (4) Duplicate studies; (5) Participants with other common eye diseases such as pathological myopia, diabetic retinopathy, and retinal artery or vein occlusion; (6) Participants suffering from low visual acuity, or inferior fixation, or with unobtainable SD-OCT images due to severe refractive interstitial opacification or severe subretinal hemorrhage; (7) Eyes of patients who had received treatment, including photocoagulation, intravitreal injection of anti-VEGF drugs, PDT, or vitrectomy.

### Data Extraction and Quality Assessment

The main elements of data extraction included author, year of publication, country or region, sample size of the PCV group and the control group, control group's disease type, gold standard, SD-OCT diagnostic strategy, detection method, device type and data items of the fourfold table: TP, FP, TN and FN.

Quality assessment was performed according to the QUADAS-2 scale, a revised version of the Quality Assessment of Diagnostic Accuracy Studies published by the QUADAS R&D team (13). This tool is comprised of four domains: patient selection, index test, reference standard, and flow and timing. Each domain is assessed for the risk of bias, and the first three domains are also evaluated for issues of applicability. The degree of risk by bias is determined as “low,” “high,” or “uncertain” based on “yes,” “no,” or “uncertain” answers to the relevant landmark questions in each section. A given domain is rated as low risk if all answers are “yes” and high risk if the answer to any informational question is “no.” A designation of “uncertain” refers to a lack of clarity in the study that makes it difficult to judge. If one answer is “uncertain,” the risk assessment is “uncertain.” One author (JY) independently assessed the quality of the literature, while the other author (SQ) independently reviewed the strategy. When the opinions of the two authors differed, the strategy was decided by open consultation.

### Statistical Analysis

Quality assessment was performed with Review Manager V5.3 (Cochrane Collaboration, London, UK) software. We performed meta-analyses using Meta-DiSc (Clinical Biostatistics Group, Hospital Ramón y Cajal, Madrid, Spain) software. First, the Spearman correlation coefficient was calculated between the logarithm of sensitivity and the logarithm of (1-specificity), and the summary receiver operating characteristic (sROC) curve was plotted. If the sROC curve shows a typical “shoulder-arm” shape, or if the correlation coefficient is significantly positive, this indicates a threshold effect of heterogeneity. The Cochran-Q test and the I^2^ statistic were further used to analyze the heterogeneity of non-threshold effect among the studies. If the included studies were not statistically heterogeneous (*P* > 0.05, I^2^ < 50%), a fixed-effects model (Mantel-Haenszel method) was used to pool the effect sizes. Otherwise, we adopted a random-effects model (DerSimonian-Laird method) to pool the effect sizes. Pooled sensitivity (Se), specificity (Sp), positive likelihood ratio (PLR), negative likelihood ratio (NLR), diagnostic odds ratio (DOR), summary receiver operating characteristic (sROC) curve, and area under the curve (AUC) were then calculated. The closer the AUC was to 1, the greater the diagnostic value. If heterogeneity was significant, a meta-regression analysis was performed to analyze the source of heterogeneity, and further subgroup analysis was performed. Fagan's plot was drawn with Stata V15.0 (StataCorp LLC, Texas, America) software to assess the clinical applicability of the diagnostic test. Sensitivity analysis was performed by sequentially omitting individual studies and calculating pooled estimates for the remaining studies to verify the stability and reliability of the results. Publication bias was assessed by Deeks' funnel plot asymmetry test.

## Results

### Literature Search

**Figure 1:**
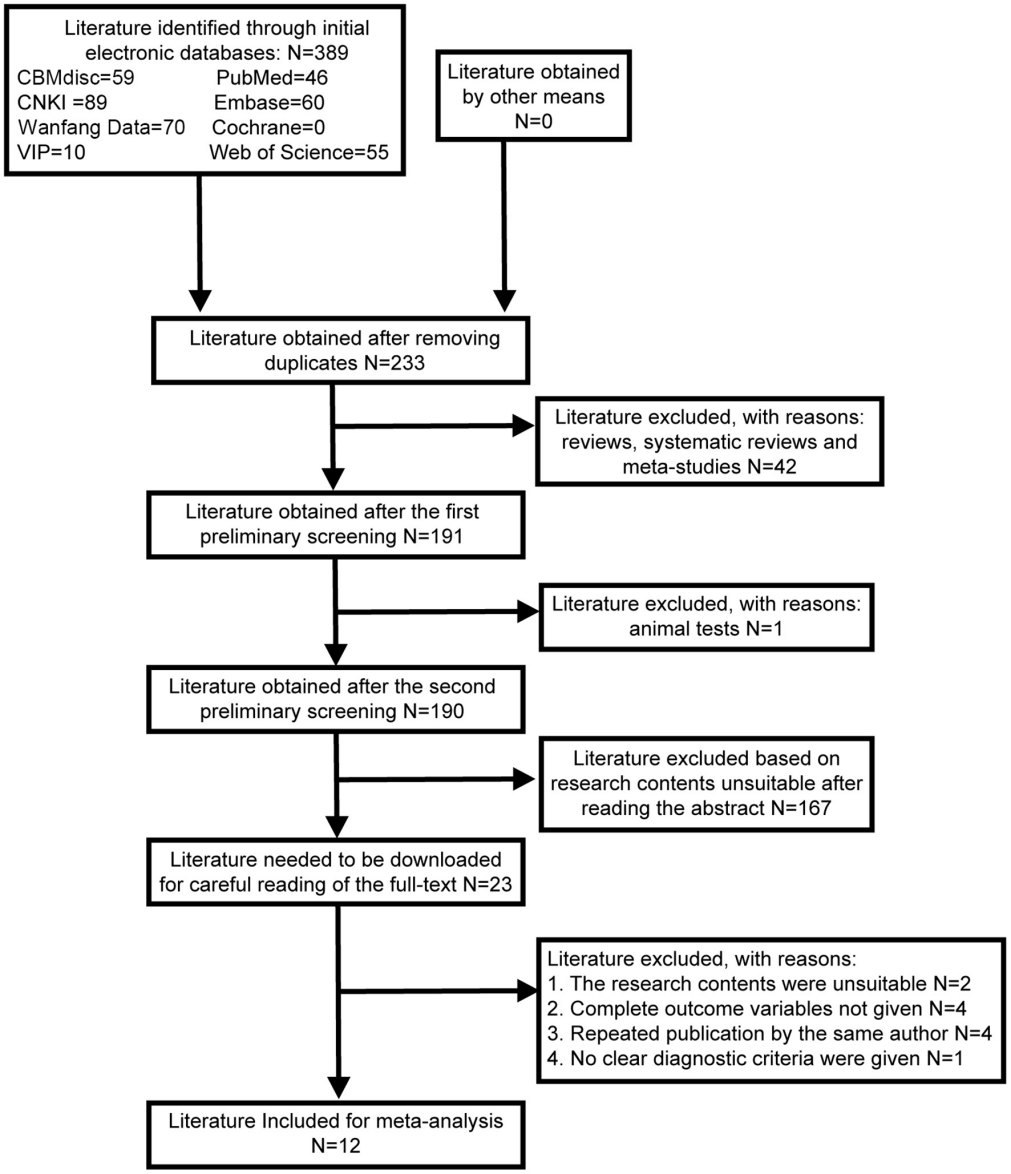
Literature retrieval flow chart.

A total of 389 studies were identified through the initial database search. After removing duplicates, reviews, systematic reviews, meta-analyses, and animal studies, 190 studies were obtained. Based on the titles and abstracts, we excluded 167 studies with inconsistent content and were left with 23 works. Then, we read the full-text versions in detail according to the eligibility criteria. Finally, 12 studies (11, 14–24) were included in the current meta-analysis, including three Chinese publications (16, 23, 24) and 9 English publications (11, 14, 15, 17–22). The PRISMA flow chart for the literature search is shown in Figure 1.

### Study Characteristics

After we comprehensively summarized the descriptive characteristics of the 12 studies, 1,348 eyes were included in our study, of which 706 eyes belonged to the PCV group and 642 eyes were in the control group (including nvAMD, dry-type AMD, CSC, serous PED, CNV, occult or idiopathic CNV and RAP). Patients in three studies (11, 20, 22) came from Caucasians, while those in 9 studies (14–19, 21, 23, 24) belonged to Asians. The descriptive characteristics of the eligible studies are presented in Table 1. The individual diagnostic strategies of the included studies are summarized in Supplementary Table 1.

**Table 1:** Characteristics of the included studies (*n* = 12).

**References**	**Country**	**Sample size (PCV/ Controls)**	**Disease type of the controls**	**Detection method**	**Gold Standard**	**Device type**	**TP**	**FP**	**TN**	**FN**
Chaikitmongkol et al. (17)	Thailand	48/71	nvAMD, CSC, RAP, serous PED	CFP+SD-OCT	ICGA	Spectralis OCT	40	12	8	59
Liu et al. (18)	China	113/75	nvAMD	SD-OCT	FFA/ICGA	Spectralis HRA+OCT	101	11	12	64
Cheung et al. (14)	Singapore	23/27	CNV, RAP	SD-OCT	FFA/ICGA	Spectralis OCT	19	13	4	14
Yang et al. (19)	China	52/51	nvAMD	CFP+ SD-OCT	FFA/ICGA	unclear	46	4	6	47
Chang et al. (15)	Korea	147/116	nvAMD	SD-OCT	ICGA	Spectralis HRA+OCT	132	18	15	98
Laíns et al. (20)	Portugal	47/53	occult CNV	SD-OCT	ICGA	Spectralis HRA+OCT and Cirrus HD-OCT	32	23	15	30
Chaikitmongkol et al. (21)	Thailand	65/59	nvAMD, CSC, RAP, idiopathic CNV	CFP+ SD-OCT	FFA/ICGA	unclear	62	3	3	56
De Salvo et al. (11)	United Kingdom	37/14	occult CNV	SD-OCT	FFA/ICGA	Spectralis HRA+OCT	35	1	2	13
Eraydin et al. (22)	Turkey	69/24	dry-type AMD, nvAMD, CSC	SD-OCT	ICGA	Spectralis HRA+OCT	52	6	17	18
Liao et al. (23)	China	24/38	wAMD	SD-OCT	FFA/ICGA	unclear	22	4	2	34
Zhang et al. (24)	China	25/38	wAMD	SD-OCT	FFA/ICGA	Cirrus HD-OCT 4000	23	4	2	34
Xia et al. (16)	China	56/76	wAMD	SD-OCT	FFA/ICGA	Spectralis HRA+OCT	49	10	7	66

*PCV, Polypoidal choroidal vasculopathy; nvAMD, neovascular age-related macular degeneration; wAMD, wet age-related macular degeneration; CSC, central serous chorioretinopathy; RAP, retinal angiomatous proliferation; CNV, choroidal neovascularization; PED, pigment epithelial detachment; ICGA, indocyanine green angiography; FFA, fundus fluorescein angiography; SD-OCT, spectral-domain optical coherence tomography; CFP, color fundus photography; Spectralis OCT, Heidelberg Engineering Inc., Heidelberg, Germany; Spectralis HRA+OCT, Heidelberg Engineering Inc., Heidelberg, Germany; Cirrus HD-OCT, Carl Zeiss Meditec Inc., Dublin, United States; Cirrus HD-OCT 4000, Carl Zeiss Meditec Inc., Dublin, United States; TP, true positives; FP, false positives; TN, true negatives; FN, false negatives*.

### Quality Assessment

**Figure 2:**
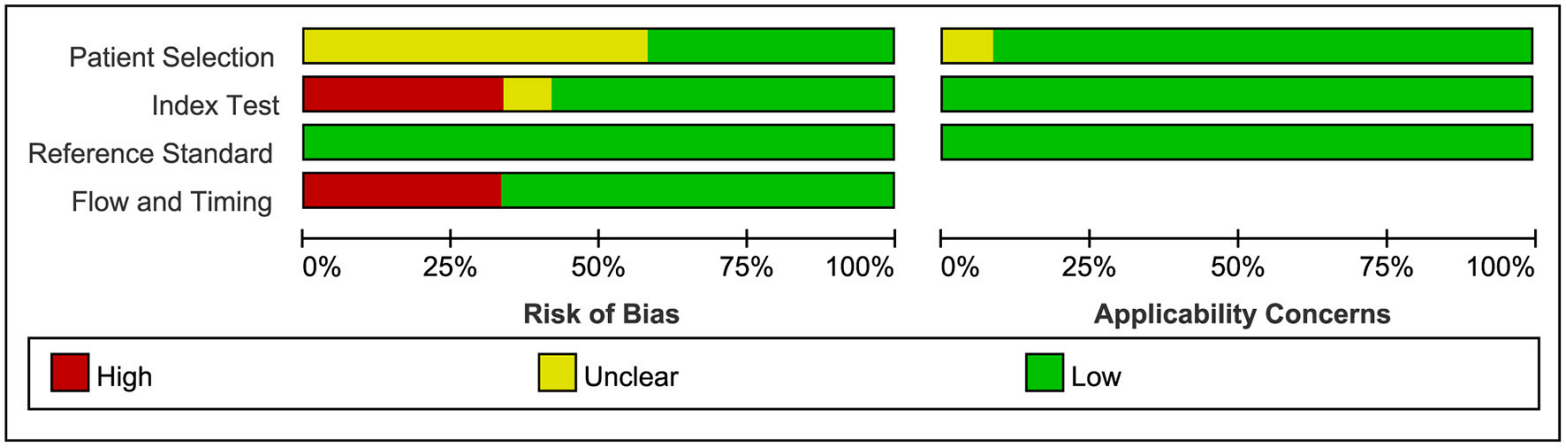
Bar chart of literature quality assessment.

**Figure 3:**
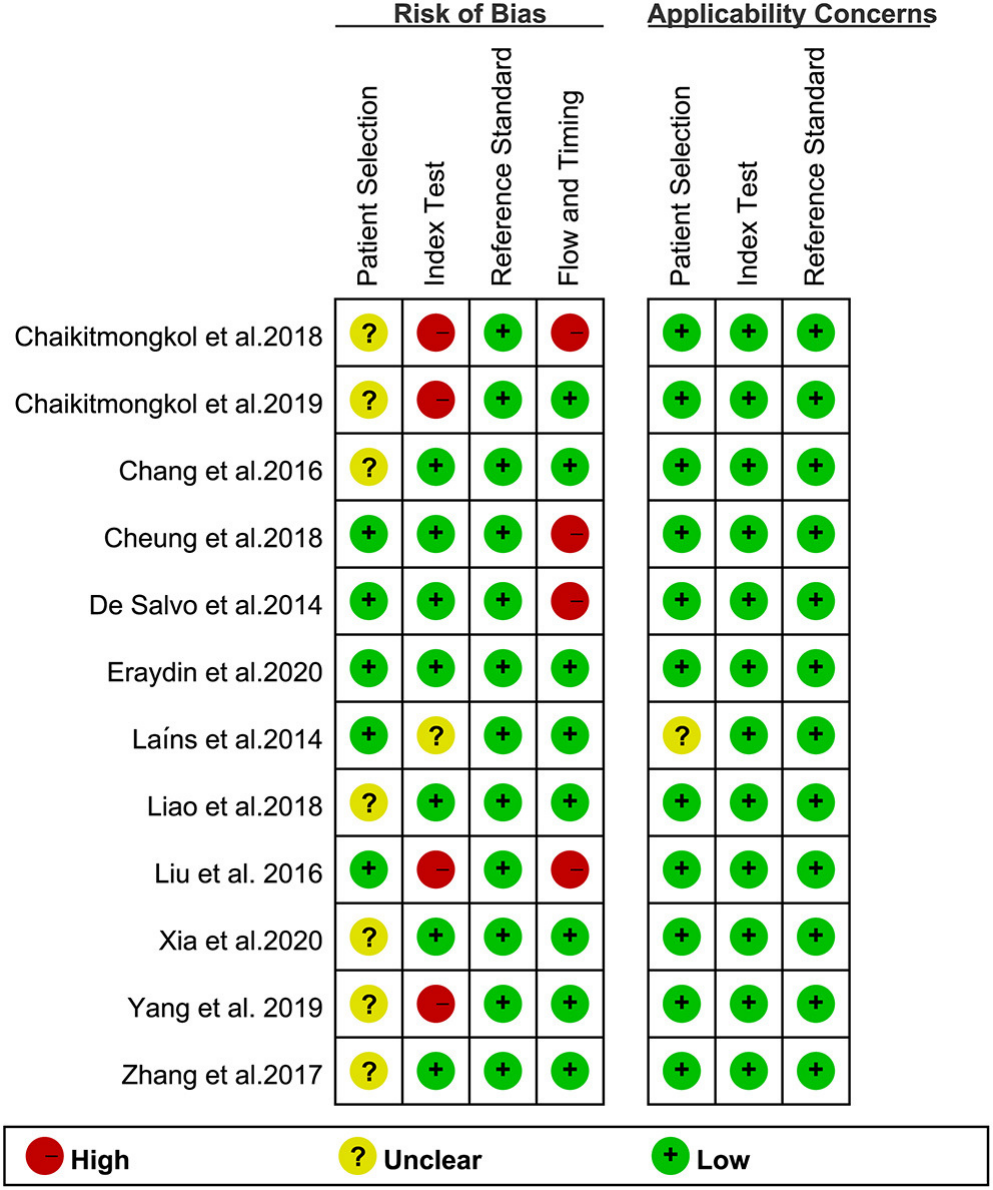
Summary of bias risk and clinical applicability. Figure 2 combined with Figure 3 together describe literature quality assessment.

The QUADAS-2 assessment of eligible studies indicated a potential risk of bias in terms of patient selection, index test, flow and timing. In terms of patient selection, seven studies were rated as “unclear” and did not specify whether the included cases were continuous or not. For the index test, since there is currently no standardized consensus OCT diagnosis strategy, seven studies adopted strategies proposed or improved by previous literature, four studies determined their strategy based on AUC ≥ 0.8, and one study had an uncertain source of the diagnostic strategy. In terms of flow and timing, not all cases met the eligibility criteria in this part because some patients did not meet the inclusion requirements, or the collection of results was incomplete. With regard to the reference standard, no bias risk was evident. Detailed descriptions of the results are shown in Figures 2, 3 and Supplementary Table 2.

### Heterogeneity Tests

The Spearman correlation coefficient between the logarithm of sensitivity and logarithm of (1-specificity) was −0.865 (*P* = 0.000 < 0.05). Simultaneously, the sROC curve showed a “shoulder-arm” shape, indicating the presence of a threshold effect in this study. The Cochran-Q test for DOR was 64.76, *P* = 0.000 < 0.05, indicating non-threshold effect heterogeneity in this study. In addition, I^2^ for Se, Sp, PLR, NLR and DOR were all 50% (*P* < 0.05 for all Cochran-Q tests), so we adopted a random-effects model to pool the five effect sizes mentioned above.

#### Assessment Criteria for Diagnostic Tests

**Figure 4:**
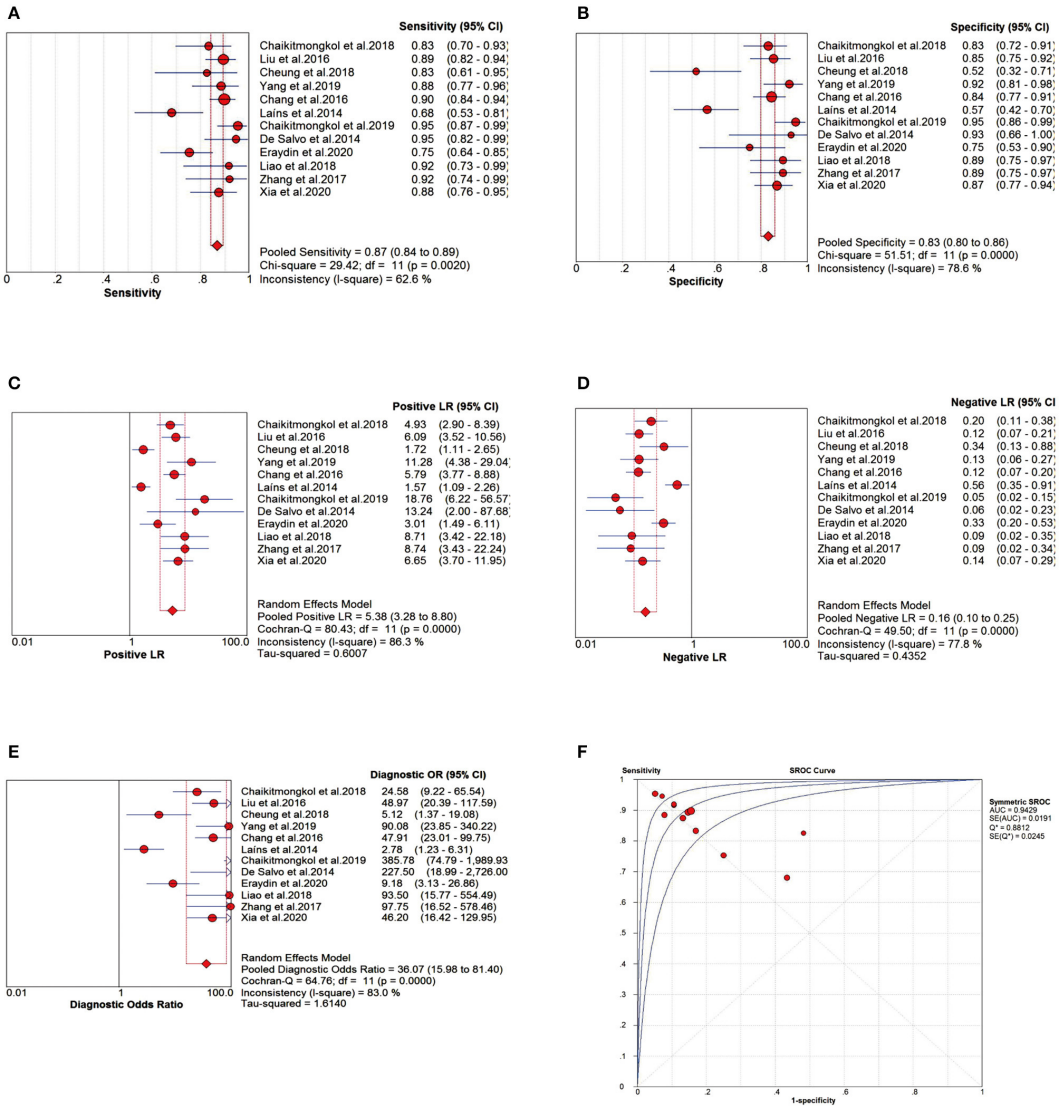
Meta-analysis forest plots of the relevant effect sizes. **(A)** The pooled sensitivity (Se). **(B)** The pooled specificity (Sp). **(C)** The pooled positive likelihood ratio (PLR). **(D)** The pooled negative likelihood ratio (NLR). **(E)** The pooled diagnostic odds ratio (DOR). **(F)** The summary receiver operating characteristic curve (sROC) and area under the curve (AUC).

The pooled sensitivity (Se) was 0.87 (95% CI: 0.84–0.89; Figure 4A), pooled specificity (Sp) was 0.83 (95% CI: 0.80–0.86; Figure 4B), pooled positive likelihood ratio (PLR) was 5.38 (95% CI: 3.28–8.80; Figure 4C), and pooled negative likelihood ratio (NLR) was 0.16 (95% CI: 0.10–0.25; Figure 4D). The pooled diagnostic odds ratio (DOR) was 36.07 (95% CI: 15.98–81.40; Figure 4E), area under the sROC curve (AUC) was 0.9429, and Q^*^ index was 0.8812 (Figure 4F).

#### Meta-Regression and Subgroup Analysis

Based on the apparent heterogeneity of non-threshold effect in this study, we extracted race [Asians, n=9 (14–19, 21, 23, 24); Caucasians, *n* = 3 (11, 20, 22)], sample size [eyes <100, *n* = 5 (11, 14, 22–24); eyes≥100, n=7 (15–21)], and device type [Spectralis HRA + OCT or Spectralis OCT, n=7 (11, 14–18, 22); others: including Cirrus HD-OCT, unknown, *n* =5 (19–21, 23, 24)] to identify the source of heterogeneity by meta-regression. The results indicated that race might be the primary source of heterogeneity in this study (RDOR=5.95, *P* = 0.0204 < 0.05; Table 2).

**Table 2:** Parameters of meta-regression analysis.

	**Coef**	**Std.Err**	***P***	**RDOR**	**95%CI**
Race	1.783	0.6346	0.0204^*^	5.95	1.42-24.99
Sample size	−0.711	0.6652	0.3165	0.49	0.11–2.28
Device type	0.238	0.6619	0.7298	1.27	0.27–6.07

* *Indicates statistical significant P-values*.

In addition, we performed the subgroup analysis according to race [Asians, *n* = 9 (14–19, 21, 23, 24); Caucasians, *n* = 3 (11, 20, 22)], detection method [SD-OCT alone, *n* = 9 (11, 14–16, 18, 20, 22–24); color fundus photography (CFP) + SD-OCT, *n* = 3 (17, 19, 21)], and disease type in controls [nvAMD only, *n* = 6 (15, 16, 18, 19, 23, 24); others: including dry-type AMD, serous PED, CSC, CNV, idiopathic or occult CNV, RAP with or without nvAMD, *n* = 6 (11, 14, 17, 20–22)]. The results indicate that the diagnostic efficacy of SD-OCT for PCV was more satisfactory in Asians than in Caucasians across all observed indexes (Se, Sp, PLR, NLR, DOR and AUC). Also, the diagnostic efficiency of SD-OCT combined with CFP was superior to that of SD-OCT alone for various observed indexes. Meanwhile, patients were divided according to the type of disease in the control group. The diagnostic accuracy of SD-OCT was better in patients with only nvAMD in the control group than in patients with other types included in the control group (Table 3).

**Table 3:** Parameters of subgroup analysis.

	**Se/95%CI**	**Sp/95%CI**	**PLR/95% CI**	**NLR /95% CI**	**DOR/95%CI**	**AUC**
**Race**						
Asians (*n* = 9)	0.89/(0.86–0.92)	0.86/(0.82–0.88)	6.28/(3.87–10.19)	0.13/(0.10–0.16)	49.01/(25.94–92.61)	0.9531
Caucasians (*n* = 3)	0.78/(0.70–0.84)	0.67/(0.56–0.77)	2.92/(1.08–7.87)	0.28/(0.12–0.67)	12.30/(2.00–75.61)	0.9021
**Detection method**						
SD–OCT (*n* = 9)	0.86/(0.83–0.89)	0.80/(0.77–0.84)	4.49/(2.59–7.79)	0.18/(0.10–0.30)	27.05/(10.47–69.89)	0.9382
SD-OCT + CFP (*n* = 3)	0.90/(0.84–0.94)	0.90/(0.84–0.94)	9.27/(3.75–22.89)	0.12/(0.06–0.25)	85.02/(18.16–398.16)	0.9622
**Disease type in controls**						
nvAMD (*n* = 6)	0.89/(0.86–0.92)	0.87/(0.83–0.90)	6.78/(5.24–8.78)	0.12/(0.09–0.16)	55.87/(36.61–85.27)	0.9488
Others (*n* = 6)	0.83/(0.78–0.87)	0.77/(0.71–0.82)	3.88/(1.82–8.28)	0.21/(0.10–0.42)	21.21/(5.26–85.54)	0.9302

*Se, sensitivity; Sp, specificity; PLR, positive likelihood ratio; NLR, negative likelihood ratio; DOR, diagnostic odds ratio; AUC, area under the sROC curve; 95%CI, 95% confidence interval; SD-OCT, spectral-domain optical coherence tomography; SD-OCT+CFP, spectral-domain optical coherence tomography combined with color fundus photography; nvAMD, neovascular age-related macular degeneration; others, inculding dry-type AMD, central serous chorioretinopathy, serous retinal pigment epithelial detachment, retinal angiomatous proliferation, choroidal neovascularization, idiopathic/occult choroidal neovascularization with and without nvAMD. n, number of included studies*.

#### Clinical Application Value

**Figure 5:**
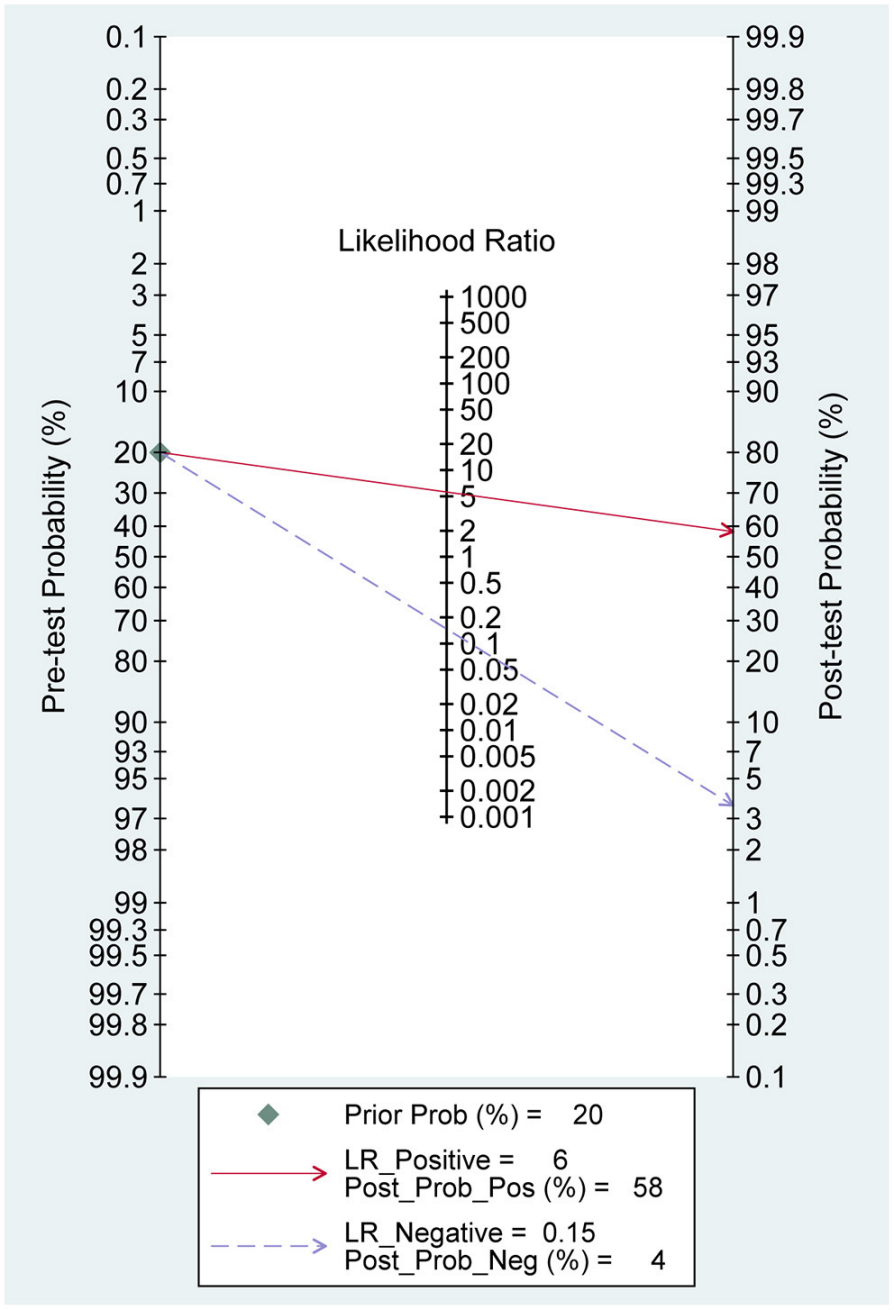
Fagan's plot.

We assumed a 20% probability of diagnosing PCV based on symptoms and ophthalmologists' personal experiences. After a positive test by SD-OCT, the accuracy of diagnosing PCV increased to 58% with reference to Fagan's plot (Figure 5). Conversely, with a negative test, the diagnostic accuracy was 4%. Therefore, the diagnosis of PCV by SD-OCT provides good accuracy and significant clinical application value.

#### Sensitivity Analysis

**Figure 6:**
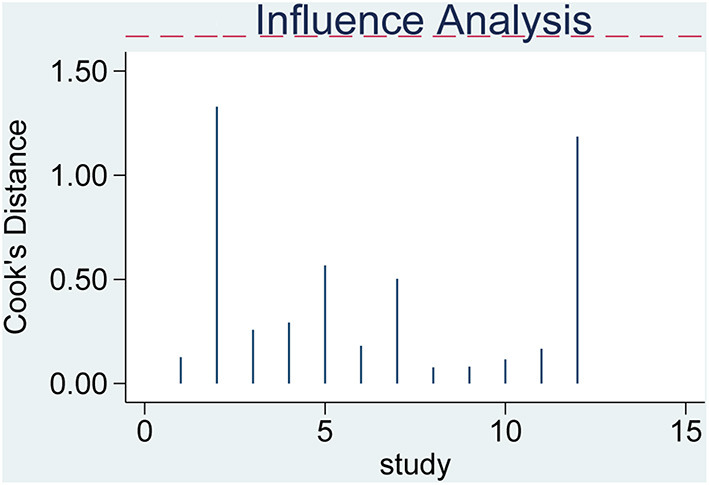
Sensitivity analysis.

A “leave-one-out” sensitivity analysis was performed by STATA V15.0. The result showed that excluding individual original studies of the current analysis one by one did not cause the calculated results to be sensitive(Figure 6).

#### Publication Bias Tests

**Figure 7:**
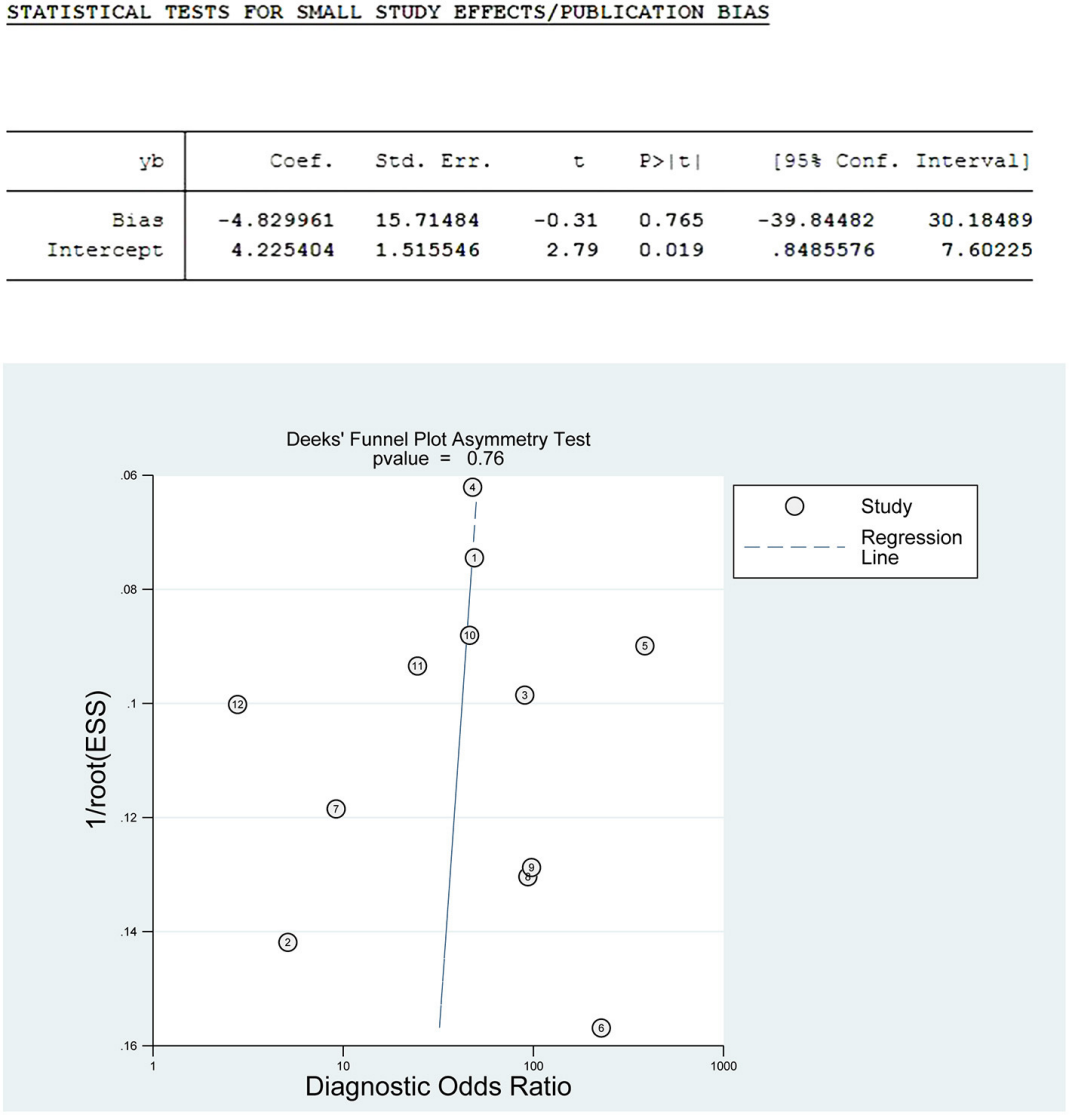
Deeks' funnel plot.

Publication bias was assessed by the Deeks' funnel plot asymmetry test with STATA V15.0. The Deeks' funnel plot had DOR as the horizontal axis and the inverse square root of the effective sample size (1/ESS) as the vertical axis. Our study showed that the regression coefficient was −4.83, *P* = 0.76 > 0.05, *t* = −0.31 (95% CI: −39.84–30.18) and the funnel plot was basically symmetrical, indicating that there was no significant publication bias in this meta-study (Figure 7).

## Discussion

This systematic assessment and meta-analysis first summarized the diagnostic value of SD-OCT for PCV and revealed that SD-OCT has high sensitivity, specificity and significant clinical application value. However, when it was negative, SD-OCT did not identify PCV well. Therefore, we recommend the combined use of color fundus photography (CFP) to improve the diagnostic efficacy.

### Characteristic Manifestations of PCV on SD-OCT

The characteristic manifestations of PCV on SD-OCT have been described in previous reports. A polypoidal lesion of PCV on SD-OCT exhibits a thumb-like retinal pigment epithelium (RPE) protrusion or a sharp peak PED (17, 25), as well as a hyporeflective halo surrounded by a hyperreflective ring underneath the PED (15, 18). The abnormal BVNs and exudation can superficially detach the RPE and Bruch's membrane, forming a “double-layer” sign on SD-OCT images (25, 26). A notch-like lesion at the edge of the PED presents a “V”-shaped depression between the two PEDs, which is associated with CNV, especially occult CNV (27). In addition, multiple PEDs and hyperreflective intraretinal hard exudates can also be observed on SD-OCT (11, 16). Although these features are not unique to PCV, many similar studies (11, 15, 16) have demonstrated statistically significant differences in the proportion of these features in patients with PCV and nvAMD, making it possible to diagnose PCV on SD-OCT.

### Interpretation of Meta-Analysis Results

This meta-analysis showed that the sensitivity and specificity of SD-OCT to diagnose PCV were high, 87% and 83%, respectively, which is consistent with previous studies. De Salvo et al. (11) first utilized SD-OCT to diagnose PCV in 2014 with the following findings: multiple PEDs, a sharp PED peak, a PED notch, a visible hyporeflective lumen within hyperreflective lesions adherent to the outer surface of the retinal pigment epithelium, and hard exudates. At least three of these features were used to diagnose with a sensitivity of 94.6% and a specificity of 92.9%. Based on De Salvo's diagnostic strategy, Xia et al. (16) added a “double-layer” sign representing the abnormal BVNs, and the sensitivity and specificity were 87.5% and 86.8%, respectively. Since PCV is a choroidal thickening disease (28–31), Chang et al. (15) recently adopted the additional criterion of subfoveal choroidal thickness 300 um. They found that the sensitivity increased from 85.7% to 89.8%, while the specificity decreased from 86.2 to 84.5%.

In contrast, the sensitivity and specificity of our results presented slightly reduced because the literature we enrolled in covered a broader selection of samples. In addition to nvAMD, our control group included other diseases such as CSC, idiopathic or occult CNV and RAP. Our study has greater clinical significance since macular hemorrhage, intraretinal hard exudates, or shallow detachment of the macular neuroepithelial layer accompanying the above diseases can also severely impair patients' vision. According to subgroup analysis, we found that for all test indexes (Se, Sp, PLR, NLR, DOR, and AUC), the diagnostic accuracy of SD-OCT was superior for the group with only nvAMD included in the control group than for other types of disease included in the control group. That resulted in a slight decrease in detection accuracy after the overall combination. Furthermore, PCV is more prevalent in Asians, accounting for 22.3–61.6% of patients with suspected nvAMD diagnosed by ICGA, compared to approximately 8–13% in Caucasians (1, 2), which slightly decreased the PCV detection rate by SD-OCT in this study.

The DOR can objectively reflect the diagnostic value of a diagnostic test. When DOR > 1, the higher the value, the better the discrimination of the test; when the value is <1, a normal person is more likely to be judged positive than a true case; when the value is 1, it means that the test cannot distinguish a normal person from a case. The DOR value in our study was 36.07 (95% CI: 15.98–81.40), which is very satisfactory, indicating that SD-OCT has a high diagnostic value for PCV. To assess the accuracy of the sROC curve, we also calculated the Q^*^ index. The larger the index, the closer the AUC is to 1, indicating the higher accuracy of a diagnostic test. In the present study, the AUC was 0.9429, and the Q^*^ index was 0.8812, indicating that SD-OCT has high diagnostic accuracy.

The PLR and NLR are not affected by prevalence in the test population and are relatively independent clinically meaningful indicators for assessing the effectiveness of diagnostic tests. The likelihood of identifying or excluding a specific disease is greatly increased when PLR > 10 or NLR < 0.1. Our study indicated the pooled PLR and NLR were 5.38 and 0.16, respectively, suggesting that SD-OCT does not identify PCV well in the presence of negative results. To improve the diagnostic efficacy, we recommend combining SD-OCT with color fundus photography (CFP). Cackett et al. (32) found that serosanguinous PEDs were more common in eyes with PCV than CNV (PCV vs. CNV = 45.7 vs. 3.9%) in Chinese patients. The drusen are often peripapillary in Caucasians but rare in colored individuals with frequently located in the macular area (33). Furthermore, a Japanese PCV Study Group (34) suggested that definitive diagnostic criteria for PCV should satisfy at least one of these findings: (1) Fundus examination shows protruded elevated orange-red lesions. (2) characteristic polypoidal lesions are seen in ICGA findings. In suspected cases, abnormal BVNs in ICGA or recurrent serous or serosanguinous PED can be observed. Thus characteristic manifestations by fundoscopy can provide ophthalmologists with clues for PCV.

Compared with traditional fundoscopy, the use of CFP does not require mydriasis and can avoid the risk of drug-induced glaucoma. It also provides a digital image, which is objective and reliable, easy to store and transmit, and can be used for long-distance diagnosis. In addition, the image capture process is fast, less costly, and has high patient compliance, which is why CFP is widely utilized in small or medium-sized medical institutions in developing countries. We analyzed the diagnostic efficacy of SD-OCT combined with CFP as a detection method. According to the subgroup analysis, the PLR increased from 4.49 to 9.27 and NLR decreased from 0.18 to 0.12 in the SD-OCT combined with CFP group compared with the SD-OCT alone group. This result indicated that SD-OCT combined with CFP could improve the diagnostic and differential efficiency (Table 3). Simultaneously, all diagnostic indicators (Se, Sp, PLR, NLR and AUC) were higher in the SD-OCT combined with CFP group (Table 3). Furthermore, Chaikitmongkol et al. (17) found that the accuracy of SD-OCT in diagnosing PCV varied in different prevalent regions due to the different clinical experience and background knowledge of ophthalmologists. However, this difference was not seen in the SD-OCT combined with CFP group, indicating that the diagnosis of SD-OCT combined with CFP is stable. In conclusion, we recommend combing SD-OCT with CFP to diagnose PCV, especially in the absence of ICGA in developing countries.

PCV has a bilateral tendency. The presence of PCV in one eye implies an increased risk of similar clinical changes in the other eye, especially in Caucasians (7, 33). However, ICGA is an invasive test, and it is not realistic to monitor the contralateral eye. In our study, the probability of diagnosing PCV after a positive test by SD-OCT increased to 58%, compared with the pre-test probability set at 20%, which confirms the significant value of SD-OCT in clinical applications. Meanwhile, SD-OCT is non-invasive, convenient, and can be repeated in a follow-up mode. Therefore, SD-OCT examination can be routinely applied to monitor the progression of PCV patients.

Sensitivity analysis is an important indicator to test the stability of a meta-analysis. Our current meta-analysis's robustness was relatively stable by sequentially omitting individual studies. Deeks' publication bias test is based on linear regression to detect whether the funnel plot is symmetrical. The slope of the regression line (regression coefficient) is equal to 0, indicating that the funnel plot is entirely symmetrical. The regression coefficient of our study was −4.83, with *P* = 0.76 > 0.05, *t* = −0.31 (95% CI: −39.84–30.18), and the studies enrolled in this paper were basically symmetrically distributed around the centerline of the funnel plot, so it was concluded that there was no significant publication bias in the current study. In summary, the results of this study are stable and reliable.

### Sources of Heterogeneity

The high heterogeneity for our study was caused by both threshold and non-threshold effects. Due to the current lack of a standardized or conventional assessment strategy for SD-OCT, different diagnostic criteria were adopted in individual original studies, thus contributing to the heterogeneity of threshold effect. After summarizing the characteristics of previous literature, we suggest that the SD-OCT diagnostic strategy for PCV should at least include the following findings. (1) Fundus examination shows retinal orange-red elevated lesions. (2) Single or multiple PEDs, a sharp PED peak, PED notches, “double-layer” signs, or a hyporeflective lumen underneath the PED representing the polyp lesion should be observed on SD-OCT. (3) If necessary, the measurement of subfoveal choroidal thickness can be added to the diagnosis.

In addition to the threshold effect, differences in test population, sample size, and test conditions probably contributed to the non-threshold heterogeneity in this study. Therefore, we integrated factors such as race, sample size, and SD-OCT device type into the meta-regression analysis. The results showed that race might be the primary source of non-threshold effect heterogeneity (*P* < 0.05). RDOR=5.95 indicated that the diagnostic accuracy of SD-OCT was 5.08 times higher in Asians than in Caucasians. Several studies have shown that 92% of PCV lesions in Asians occur in the macula (7), while in Caucasians, PCV is more common outside the macula, including around the optic disc and in the peripheral fundus (2, 33, 35). In general, fundus scans on SD-OCT are performed at 30° around the posterior pole, where most maculopathies are located. When PCV lesions are situated away from the macular area or in the peripheral fundus, it is difficult for SD-OCT to detect abnormal lesions.

### Limitations

Nevertheless, several other limitations were encountered in this review study. First, the number of cases was limited due to the low prevalence of PCV in the population. Second, our study only covered English and Chinese literature. Although the publication bias of this paper was not significant, the language potentially restricted the quality of our article. Finally, this study only included naïve PCV patients. Whether treatment can reduce the diagnostic efficiency of SD-OCT for PCV remains unknown.

## Conclusion

In conclusion, our results suggest that SD-OCT has high sensitivity, specificity, and significant clinical applicability for differentiating PCV from diseases such as nvAMD that tend to cause serous or serosanguinous retinal pigment epithelial detachment. Since color fundus photography (CFP) is more accessible in the clinic and can improve the diagnostic efficacy of SD-OCT, we recommend combining SD-OCT with CFP, especially without ICGA.

## Data Availability Statement

The original contributions presented in the study are included in the article/Supplementary Materials. Further inquiries can be directed to the corresponding author/s.

## Author Contributions

YJ: concept and design, data analysis, and preliminary draft writing. SQ: participation in manuscript writing and editing. YJ and SQ: literature retrieval, data extraction, and quality assessment. All authors contributed to the article and approved the submitted version.

## Conflict of Interest

The authors declare that the research was conducted in the absence of any commercial or financial relationships that could be construed as a potential conflict of interest.

## Publisher's Note

All claims expressed in this article are solely those of the authors and do not necessarily represent those of their affiliated organizations, or those of the publisher, the editors and the reviewers. Any product that may be evaluated in this article, or claim that may be made by its manufacturer, is not guaranteed or endorsed by the publisher.
