# Supplementary material for: Diagnostic Value of Spectral-Domain Optical Coherence Tomography for Polypoidal Choroidal Vasculopathy: A Systematic Review and Meta-Analysis
Source: Front Med (Lausanne). 2022 Jun 15;9:878946. doi: 10.3389/fmed.2022.878946 (PMC9242399; doi:10.3389/fmed.2022.878946)
Supplement: Supplementary file 1 [file Data_Sheet_1.docx]

***Supplementary Material***

1. **List of Captions**

| **Captions** | **Contents** |
| --- | --- |
| Table 1 | The individual diagnostic strategies of SD-OCT for the included studies |
| Table 2 | QUADAS-2 assessment of risk of bias |
| Appendix | Search terms |

1. **Tables**

**Table1** Individual diagnostic strategies of SD-OCT for the included studies (n=12).

| **Author, Year** | **Diagnostic strategies for SD-OCT** |
| --- | --- |
| Chaikitmongkol et al. 2018 | The following features are examples of clinical cues for PCV:   - CFP: subretinal orange nodules, hemorrhagic or fibrovascular PEDs, massive subretinal hemorrhage, peripapillary location, multifocal lesions, or no large drusen in the fellow eye. - OCT: sharply peaked RPE protrusion, notched PED, hyperreflective ring surrounding hyporeflective halo underneath the PED, or DLS. |
| Liu et al. 2016 | The OCT-based diagnosis of PCV was based on the presence of at least two of the following three criteria: single or multiple dome-shaped PEDs, DLS, the TLPs were defined as positive if at least one of the following three conditions was present: sharply peaked RPE protrusions, an underlying hyperreflective ring surrounding an area of hyporeflectivity, or notch-like lesions at the margin of the PED. |
| Cheung et al. 2018 | The OCT-based diagnosis of PCV was made in the presence of two of the following three criteria: notched/narrow-peaked PED, round sub-RPE hyporeflective lesion, any RPE detachment (including PED/DLS). |
| Yang et al.2019 | The OCT-based PCV was diagnosed with at least two of the following five criteria:   - CFP: subretinal orange nodules. - OCT: thumb-like PED, notched PED, bubble sign, Bruch's membrane depression under serosanguinous PED. |
| Chang et al.2016 | - Strategy 1: The OCT-based diagnosis of PCV was based on the presence of at least three of the following findings: multiple PEDs, a sharp PED peak, PED notch, a rounded hyporeflective area representing the polyp lumen within hyperreﬂective lesions adhered beneath the retinal pigment epithelium, the presence of hyperreﬂective intraretinal hard exudates. - Strategy 2*: If the subfoveal choroidal thickness was greater than 300 um, the eye was considered PCV despite displaying only two OCT features. |
| Laíns et al.2014 | The OCT findings which were most significantly associated with providing a correct PCV diagnosis were the identification of subfoveal and extrafoveal polypoidal lesions and a notch at the margin of serous retinal pigment epithelium detachment. |
| Chaikitmongkol et al. 2019 | The OCT-based diagnosis of PCV was made in the presence of two of the following four criteria:   - CFP: a notched or hemorrhagic PED. - OCT: a sharply peaked PED at an angle of 70° to 90°, a notched or multilobulated PED, a hyperreflective ring underneath PED. |
| De Salvo et al.2014 | PCV was diagnosed based on the coexistence of at least three of the following SD-OCT findings: a sharp PED peak, a PED notch, a visible hyporeflective lumen within hyperreflective lesions adherent to the outer surface of the retinal pigment epithelium, multiple PEDs, hard exudates. |
| Eraydin et al.2020 | The OCT-based PCV was diagnosed with at least three of these findings: multiple PEDs, sharp PED peak, PED notch, hyporeflective lumen representing polyps, DLS, intraretinal hard exudates. |
| Liao et al. 2018 | - Strategy 1: The OCT-based PCV diagnosis was based on three of the following six findings: sharp PED peak, DLS, multiple PEDs, PED notch, hyporeflective lumen representing polyps, intraretinal hard exudates. - Strategy 2*: The sharp PED peak and DLS as the essential criteria, and the other features as the secondary conditions: PCV could be identified if the patient met the two essential and one of the secondary conditions. Or they didn't meet the essential conditions, but three secondary conditions could also diagnose PCV. |
| Zhang et al. 2017 | - Strategy 1: The OCT-based PCV diagnosis was based on at least three of the following six findings: sharp PED peak, DLS, multiple PEDs, PED notch, hyporeflective lumen representing polyps, intraretinal hard exudates. - Strategy 2*: The sharp PED peak and DLS as the essential criteria, and the other features as the secondary conditions: when either the sharp PED peak or DLS existed, PCV diagnosis based on at least one of the secondary features. When neither the sharp PED peak nor DLS existed, the diagnosis of PCV should be made at least three secondary elements. |
| Xia et al.2020 | The OCT-based PCV was diagnosed with at least three of the following findings: single or multiple PEDs, sharp PED peak, PED notch, hyporeflective lumen representing polyps, DLS. |

*PCV, Polypoidal choroidal vasculopathy; PED, pigment epithelial detachment; DLS, double-layer sign; OCT, optical coherence tomography; CFP, color fundus photography.*

** When the literature provided two diagnostic strategies, the diagnostic strategy we adopted.*

**Table2** QUADAS-2 assessment of risk of bias (n=12).

| **Author, year** | **Risk of bias** | | | | | **Applicability concerns** | |
| --- | --- | --- | --- | --- | --- | --- | --- |
|  | **Patient selection** | **Index test** | **Reference standard** | **Flow and timing** | **Patient selection** | **Index test** | **Reference standard** |
| Chaikitmongkol et al. 2018 | Unclear | High | Low | High | Low | Low | Low |
| Liu et al. 2016 | Low | High | Low | High | Low | Low | Low |
| Cheung et al. 2018 | Low | Low | Low | High | Low | Low | Low |
| Yang et al.2019 | Unclear | High | Low | Low | Low | Low | Low |
| Chang et al.2016 | Unclear | Low | Low | Low | Low | Low | Low |
| Laíns et al.2014 | Low | Unclear | Low | Low | Unclear | Low | Low |
| Chaikitmongkol et al. 2019 | Unclear | High | Low | Low | Low | Low | Low |
| De Salvo et al.2014 | Low | Low | Low | High | Low | Low | Low |
| Eraydin et al.2020 | Low | Low | Low | Low | Low | Low | Low |
| Liao et al. 2018 | Unclear | Low | Low | Low | Low | Low | Low |
| Zhang et al. 2017 | Unclear | Low | Low | Low | Low | Low | Low |
| Xia et al.2020 | Unclear | Low | Low | Low | Low | Low | Low |

*QUADAS-2, Quality Assessment of Diagnostic Accuracy Studies-2.*

1. **Search terms**

**3.1 English publications**

- search terms through PubMed：

Search: (((polypoidal choroidal vasculopathy[Title/Abstract]) OR (PCV[Title/Abstract])) AND ((((((((Tomography, Optical Coherence[Title/Abstract]) OR (Coherence Tomography, Optical[Title/Abstract])) OR (OCT Tomography[Title/Abstract])) OR (Tomography, OCT[Title/Abstract])) OR (Optical Coherence Tomography[Title/Abstract])) OR (spectral-domain optical coherence tomography[Title/Abstract])) OR (spectral domain optical coherence tomography[Title/Abstract])) OR (SD-OCT[Title/Abstract]))) AND (sensitiv*[Title/Abstract] OR sensitivity and specificity[MeSH Terms] OR (predictive[Title/Abstract] AND value*[Title/Abstract]) OR predictive value of tests[MeSH Term] OR accuracy*[Title/Abstract])Filters: from 2010/1/1 - 2021/7/9

- search terms through Embase：

#9 #3 AND #6 AND #7 AND #8

#8 [2010-2021]/py

#7 ' sensitiv ': ab , ti OR ' sensitivity and specificity ': ab , ti OR ' predictive ': ab , ti OR ' predictive value of tests ': ab , ti OR' accuracy ': ab , ti

#6 #4 OR #5

#5 ' coherence tomography , optical ': ab , ti OR ' oct tomography ': ab , ti OR ' tomography , oct ': ab ,ti OR ' optical coherence tomography ': ab , ti OR 'spectral - domain optical coherence tomography' : ab , ti OR 'spectral domain optical coherence tomography ': ab , ti OR ' sd - oct' : ab , ti

#4 tomography, AND optical AND coherence

#3 #1OR #2

#2 ' pcv ': ab , ti

#1 polypoidal AND choroidal AND (' vasculopathy '/ exp OR vasculopathy )

- search terms through Cochrane：

#1 polypoidal choroidal vasculopathy

#2 ( PCV ): ab , ti , kw

#3 #1 OR #2

#4 Tomography , Optical Coherence

#5 ( Coherence Tomography , Optical ): ab , ti , kw OR (ОCT Tomography ):ab , ti , kw OR ( Tomography ,ОСТ): ab , ti , kw OR ( Optical Coherence Tomography ): ab , ti , kw OR ( spectral - domain optical coherence tomography ): ab , ti , kw OR ( spectral domain optical coherence tomography ): ab , ti , kw OR ( SD -ОСТ): ab , ti , kw

#6 #4OR#5

#7 ( sensitiv ): ab , ti , kw OR ( sensitivity and specificity ): ab , ti , kw OR ( predictive ): ab , ti , kw OR ( predictive value of tests ): ab , ti , kw OR ( accuracy ): ab , ti , kw

#8 #3AND #6AND#7

- search terms through Web of Science

TS=(Tomography, Optical Coherence or Coherence Tomography, Optical or OCT Tomography or Tomography, OCT or Optical Coherence Tomography or spectral-domain optical coherence tomography or spectral domain optical coherence tomography or SD-OCT) AND TS=(polypoidal choroidal vasculopathy or PCV) AND TS=(sensitive or sensitivity and specificity or predictive or predictive value of tests or accuracy )

**3.2 Chinese publications**

- 万方检索式(Wanfang)：

(主题:(息肉状脉络膜血管病变 OR PCV) and 主题:(光学相干断层扫描 OR 频域光相干断层扫描 OR 频域光学相干断层扫描 OR 光相干断层扫描 OR OCT OR 频域OCT OR SD-OCT) and 主题:(诊断)) and Date:2010-*

- 维普检索式(VIP)：

题名或关键词=息肉状脉络膜血管病变+ PCV AND 题名或关键词=光学相干断层扫描+频域光相干断层扫描+频域光学相干断层扫描+光相干断层扫描+ OCT +频域 OCT + SD-OCT AND 文摘=诊断 AND 年份:2010-2021

- 中国知网检索式 (CNKI)：

（主题：息肉状脉络膜血管病变） OR （主题： PCV ) AND (（主题：光学相干断层扫描） OR （主题：频域光相干断层扫描） OR （主题：频域光学相干断层扫描） OR （主题：光相干断层扫描） OR （主题： OCT ) OR （主题：频域 OC T ) OR （主题： SD -OCT)) AND (（摘要：诊断）)

- CBM检索式：

#9 ((#7) AND (#6) AND (#3)) AND 2010-2021[日期]

#8 (#7) AND (#6) AND (#3)

#7 诊断

#6 (#5) OR (#4)

#5 "频域光相干断层扫描"[常用字段:智能] OR "频域光学相干断层扫描"[常用字段:智能] OR "光相干断层扫描"[常用字段:智能] OR "OCT"[常用字段:智能] OR "频域OCT"[常用字段:智能] OR "SD-OCT"[常用字段:智能]

#4 光学相干断层扫描

#3 (#2) OR (#1)

#2 PCV

#1 息肉状脉络膜血管病变
